# Combining TMEM Doorway Score and Mena^Calc^ Score Improves the Prediction of Distant Recurrence Risk in HR+/HER2− Breast Cancer Patients

**DOI:** 10.3390/cancers14092168

**Published:** 2022-04-26

**Authors:** Xianjun Ye, Maja H. Oktay, Xiaonan Xue, Thomas E. Rohan, Paula S. Ginter, Timothy D’Alfonso, Elizabeth N. Kornaga, Don G. Morris, David Entenberg, John S. Condeelis

**Affiliations:** 1 Department of Anatomy and Structural Biology, Albert Einstein College of Medicine/Montefiore Medical Center, Bronx, NY 10461, USA; xianjun.ye@einsteinmed.edu (X.Y.); moktay@montefiore.org (M.H.O.); 2 Gruss-Lipper Biophotonics Center, Albert Einstein College of Medicine/Montefiore Medical Center, Bronx, NY 10461, USA; 3 Integrated Imaging Program, Albert Einstein College of Medicine/Montefiore Medical Center, Bronx, NY 10461, USA; 4 Department of Pathology, Albert Einstein College of Medicine/Montefiore Medical Center, Bronx, NY 10461, USA; 5 Department of Epidemiology and Population Health, Albert Einstein College of Medicine/Montefiore Medical Center, Bronx, NY 10461, USA; xiaonan.xue@einsteinmed.edu (X.X.); thomas.rohan@einsteinmed.edu (T.E.R.); 6 Department of Pathology, NYU Langone Hospital-Long Island, Mineola, NY 11501, USA; paula.ginter@nyulangone.org; 7 Department of Pathology, Memorial Sloan Kettering Cancer Center, New York, NY 10021, USA; dalfonst@mskcc.org; 8 Translational Laboratories, Tom Baker Cancer Centre, Calgary, AB T2N 4N2, Canada; elizabeth.kornaga@albertahealthservices.ca (E.N.K.); donald.morris@albertahealthservices.ca (D.G.M.); 9 Department of Oncology, University of Calgary, Calgary, AB T2N 4N2, Canada; 10 Department of Cell Biology, Albert Einstein College of Medicine/Montefiore Medical Center, Bronx, NY 10461, USA; 11 Department of Surgery, Albert Einstein College of Medicine/Montefiore Medical Center, Bronx, NY 10461, USA

**Keywords:** TMEM doorway, Mena^Calc^, metastasis, prognostic, combined marker, RMST difference

## Abstract

**Simple Summary:**

90% of breast cancer mortality is caused by distant metastasis, a process that involves both dissemination of cancer cells to distant sites as well as their proliferation after arrival. However, prognostic assays currently used in the clinic are based on proliferation and do not measure tumor cell dissemination potential. We previously reported that the density of Tumor Microenvironment of Metastasis (TMEM) doorways (portals for cancer cell intravasation and dissemination) is a prognostic biomarker for the development of distant metastasis in hormone receptor positive/human epidermal growth factor receptor 2 negative (HR+/HER2−) patients. We have shown further that Mena^Calc^, a mechanistically linked (but independent) biomarker for distant metastasis, is prognostic in some cohorts of triple-negative patients. Here, we develop and compare several digital pathology-based machine vision algorithms to investigate if a combined TMEM-Mena^Calc^ biomarker could provide improved prognostic information over and above that of either biomarker alone.

**Abstract:**

Purpose: to develop several digital pathology-based machine vision algorithms for combining TMEM and Mena^Calc^ scores and determine if a combination of these biomarkers improves the ability to predict development of distant metastasis over and above that of either biomarker alone. Methods: This retrospective study included a subset of 130 patients (65 patients with no recurrence and 65 patients with a recurrence at 5 years) from the Calgary Tamoxifen cohort of breast cancer patients. Patients had confirmed invasive breast cancer and received adjuvant tamoxifen therapy. Of the 130 patients, 86 cases were suitable for analysis in this study. Sequential sections of formalin-fixed paraffin-embedded patient samples were stained for TMEM doorways (immunohistochemistry triple staining) and Mena^Calc^ (immunofluorescence staining). Stained sections were imaged, aligned, and then scored for TMEM doorways and Mena^Calc^. Different ways of combining TMEM doorway and Mena^Calc^ scores were evaluated and compared to identify the best performing combined marker by using the restricted mean survival time (RMST) difference method. Results: the best performing combined marker gave an RMST difference of 5.27 years (95% CI: 1.71–8.37), compared to 3.56 years (95% CI: 0.95–6.1) for the associated standalone TMEM doorway analysis and 2.94 years (95% CI: 0.25–5.87) for the associated standalone Mena^Calc^ analysis. Conclusions: combining TMEM doorway and Mena^Calc^ scores as a new biomarker improves prognostication over that observed with TMEM doorway or Mena^Calc^ Score alone in this cohort of 86 patients.

## 1. Introduction

Distant metastasis is the primary cause (~90%) of death from breast cancer [1]. However, current gene expression-based biomarkers for disease outcome (e.g., PAM50, Oncotype DX, MammaPrint) [2,3] are largely driven by proliferation and estrogen-regulation genes, and do not provide conclusive information about the risk of systemic dissemination with consequent distant metastasis [3,4,5].

Previously, we reported the discovery of the “Tumor Microenvironment of Metastasis” (TMEM) doorway, a portal into the blood vasculature composed of a tumor cell overexpressing the actin regulatory protein Mena, a perivascular macrophage, and an endothelial cell, all in direct contact (Figure S1A–D left,E). TMEM doorways function as vascular openings through which tumor cells intravasate and disseminate hematogenously [6,7]. We previously showed that a triple immunohistochemical stain for the three constituent cell types that make up TMEM doorways can be used as a biomarker (called TMEM Score) for prognosticating the development of distant metastasis [8]. We further showed that TMEM Score prognosticates the risk of distant metastasis in HR+/HER2− breast cancer patients better than the IHC4 immunohistochemical assay score [9] and independently of classical clinicopathologic features [4]. Finally, we analytically validated a quantification of the TMEM Score using an automated, high-throughput assay implemented within a Clinical Laboratory Improvement Amendments (CLIA) certified clinical diagnostic laboratory and showed that TMEM Score is significantly associated with early distant recurrence (within 5-years of diagnosis) [10].

While these studies clinically validated the use of TMEM Score for prognosticating metastatic outcome in HR+/HER2− patients (the largest subgroup of breast cancer patients), a statistically significant association between TMEM Score and distant recurrence outcome was not observed in the triple-negative or HER2+ breast cancer subpopulations, perhaps due to the smaller number of these subjects available for analysis. While there is currently no evidence of a connection between the HER2 receptor status and TMEM doorways or Mena^Calc^, we cannot rule out its existence.

**Figure 1:**
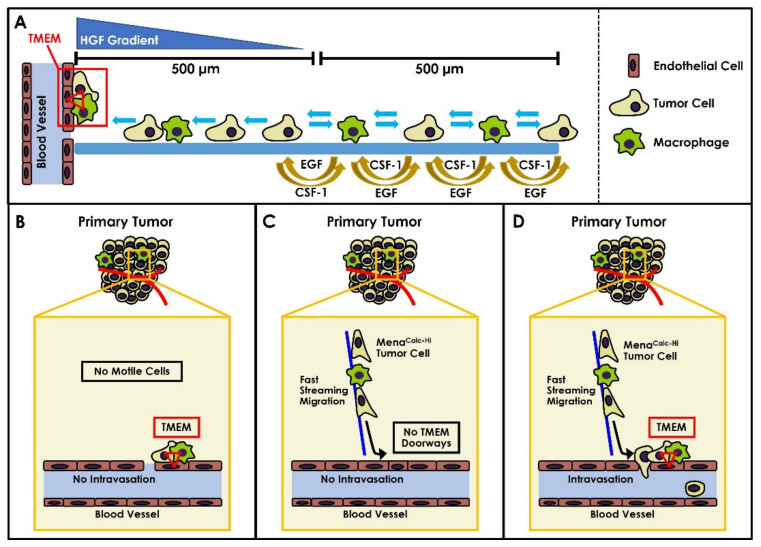
Illustration of the biological rationale leading to the TMEM-Mena^Calc^ combined marker. (**A**) Macrophages and tumor cells with an elevated Mena^Calc^ isoform expression pattern communicate with each other via a paracrine loop that enables them to co-migrate along collagen fibers towards blood vessels. Within 500 µm of blood vessels, endothelial-cell-secreted HGF gradients provide a directional chemoattractant signal to the fast-migrating tumor cells. (**B**) In regions of tumors that that contain TMEM doorways but lack Mena^Calc-Hi^ tumor cells, no intravasation would be possible. (**C**) In regions of tumors that contain Mena^Calc-Hi^ tumor cells but lack TMEM doorways, no intravasation would be possible. (**D**) In regions of tumors where there is a spatial overlap of Mena^Calc-Hi^ tumor cells and TMEM doorways, successful intravasation of tumor cells can occur.

To identify those cancer cells within the tumor that are capable of intravasation, we developed the in vivo invasion assay, a technique capable of isolating the motile fraction of cancer cells from the rest of the immobile bulk of the primary tumor [11,12,13]. Using this assay in mouse models of breast cancer, we were able to determine that a subset of tumor cells and macrophages communicate with each other via a paracrine loop that enables them to co-migrate together along collagen fibers at 10–100 times the speed of the rest of the tumor cells within the bulk tumor. This type of coordinated cellular motion is known as “fast streaming migration” [13]. We further determined that endothelial-cell-secreted Hepatocyte Growth Factor (HGF) gradients provide a directional chemoattractant signal which attracts fast-migrating cells that are less than 500 µm away from blood vessels [14] (Figure 1A). Expression profiling of these cells showed that Mena, a key actin polymerization regulatory protein, plays an important role in potentiating tumor cell motility as well as tumor cell intravasation near TMEM doorways [15,16,17].

Mena consists of several splice-variant isoforms which confer distinct phenotypes to tumor cells [18]. Of these isoforms, Mena11a, an anti-metastatic isoform that is strongly associated with an epithelial phenotype, is down-regulated during epithelial-to-mesenchymal transition (EMT) and in invasive tumor cells [18]. Several other isoforms, including Mena^INV^, have been shown to confer a pro-metastatic motile phenotype and are found to be expressed exclusively in invasive and disseminating tumor cells [19]. We have found that tumor cells that have high levels of overall Mena expression, and also contain a Mena^INV-Hi^ and Mena11a^Low^ isoform expression pattern, are involved in invasion, fast streaming migration, and intravasation [14,16,20]. Based upon these observations, we developed a quantitative immunofluorescence (IF)-based biomarker designed to quantify the relative amounts of pro-metastatic and anti-metastatic Mena isoforms. This metric, termed Mena^Calc^, is computed by quantifying the abundance of the Mena11a isoform (Figure S1A–D right) and subtracting the normalized value of this isoform from the normalized amount of PanMena (Figure S1A–D middle), i.e., all Mena isoforms present.

In initial retrospective studies, Mena^Calc^ was shown to be prognostic of distant metastasis in the ER- and in the node-positive subsets of a cohort of patients [21]. A second study in a different cohort [22] showed that Mena^Calc^ is prognostic in a node-negative subset of patients.

Given the difference in performance of TMEM doorway and Mena^Calc^ scores in patients with diverse breast cancer subtypes, we asked how we might be able to improve the prognostic ability of these tests. We reasoned that no intravasation would be possible within the tumors of patients that contain TMEM doorways, but which lack Mena^Calc-Hi^ tumor cells capable of intravasating through the TMEM doorways (Figure 1B). Similarly, no intravasation would be possible within tumors that contain fast streaming and highly invasive Mena^Calc-Hi^ tumor cells, but which lack TMEM doorways (Figure 1C). Successful intravasation of tumor cells would require both motile Mena^Calc-Hi^ tumor cells and TMEM doorways (Figure 1D). Thus, it is logical to suggest that patients with both high TMEM Score and high Mena^Calc^ Score would have higher risk of distant metastasis and a worse prognosis.

However, it is unclear from Mena^Calc^ alone which subgroup would most benefit from a combined TMEM-Mena^Calc^ biomarker. Since ER+/HER2− is the most common subtype of breast cancer with the longest time to recurrence, it is a high priority to determine if we can improve prognostication in this subtype.

Thus, our primary goal in this proof-of-principle initial study was to determine if a combined TMEM-Mena^Calc^ biomarker is able to improve upon the prognostication ability of either marker alone, all within a small cohort of patients with HR+/HER2− breast cancer (see Section 4 Materials and Methods for cohort description). Since HR+/HER2− is the most common subtype of breast cancer and has the longest time to recurrence, there is an urgent need to find better prognosticators of metastatic outcome for this subtype. Furthermore, multivariate analysis (including tumor size, grade, and nodal status) showed that TMEM doorway density is prognostic for distant recurrence in patients with ER+ breast cancer [10], independent of these clinical factors. Thus, it is of particular interest to determine if combining TMEM Score with Mena^Calc^ can improve TMEM Score performance in mixed-patient populations of the type studied previously. To accomplish this, we have evaluated several different ways of combining TMEM and Mena^Calc^ scores to create a multi-parameter quantitative analysis with much improved prognostic value for distant metastasis in breast cancer patients.

## 2. Results

### 2.1. Methods of Biomarker Combination

**Figure 2:**
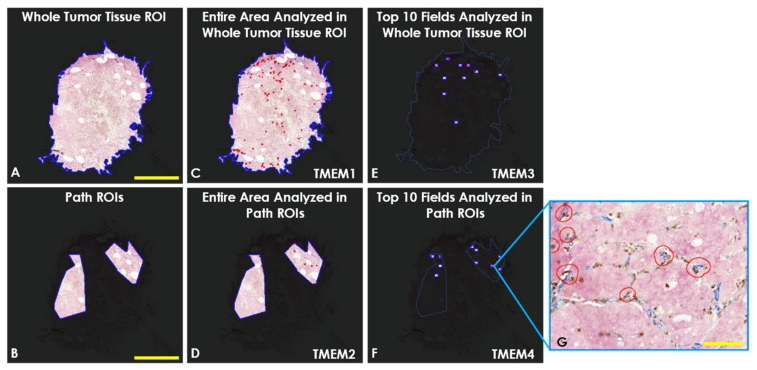
Illustration of the different types of TMEM doorway analyses performed. Four methods of TMEM doorway quantification were tested, differing in range and quantity of tissue analyzed. (**A**) Whole-Tumor Tissue Region of Interest (ROI). The entire range of the tissue is used for analysis. (**B**) Path ROIs. The analysis of the tissue is limited to a sub-region of the tissue predetermined by the pathologists as areas of invasive tumor with limited levels of stroma and immune reaction. (**C**) Entire Area Analyzed in Whole-Tumor Tissue ROI. TMEM doorway density (doorways per unit area) is quantified across entire area of the Whole Tumor Tissue ROI. This analysis is called TMEM1. (**D**) Entire Area Analyzed in Path ROIs. TMEM doorway density is quantified across entire area of Path ROIs. This analysis is called TMEM2. (**E**) Fields of View Analyzed in Whole-Tumor Tissue ROI. TMEM doorways are identified across the Whole-Tumor Tissue ROI and the TMEM Score is generated by the summing of the number of TMEM doorways within the 10 highest-scoring high-power fields of view. This analysis is called TMEM3. (**F**) Fields of View Analyzed in Path ROIs. TMEM doorways are identified across the Path ROIs and the TMEM Score is generated by the summing of the number of TMEM doorways within the 10 highest-scoring high-power fields of view. This analysis is called TMEM4. Red dots in C-F represent identified TMEM doorways. (**G**) Single high-power field. Field of View = 330 × 440 µm^2^. Red outlies contain identified TMEM doorways. Scale bars in (**A**–**F**) = 5 mm. Scale bar in (**G**) = 100 µm.

All analyses performed in this study used our previously published automated TMEM doorway identification and quantification algorithm [5,10] with variation in ROI type and tissue coverage, defined as follows: First, this study varied the range of tissue analyzed, i.e., analyses either spanned a region of interest (ROI) that contained the entire tumor tissue (Whole Tumor Tissue ROI, Figure 2A) or was limited to a sub-region of the most representative tumor tissue predetermined by the pathologists (Path ROIs, Figure 2B). Second, TMEM doorway density (doorways per unit area) was measured either within the entire ROIs (“Entire Area”, Figure 2C,D) or in the 10 high-power fields of view containing the highest TMEM doorway density measured within the ROIs (“Top 10 Fields”, Figure 2E,F). Thus, in total, four TMEM doorway quantification methods were tested. An example field of view showing identified TMEM doorways is presented in Figure 2G.

The first method (TMEM1) scored TMEM doorway density across the entire tissue area within the Whole Tumor Tissue ROI (Figure 2C). The second method (TMEM2) scored the TMEM doorway density within the Path ROIs (Figure 2D). The third (TMEM3) and fourth (TMEM4) methods quantified TMEM doorway density, as described previously [10], as the sum of TMEM doorways within a given area: namely, the 10 high power fields of view (40× magnification, 330 × 440 µm^2^) that contained the most TMEM doorways in either the Whole Tumor Tissue ROI (Figure 2E) or the Path ROIs (Figure 2F), respectively. These different methods are summarized in Table 1.

**Figure 3:**
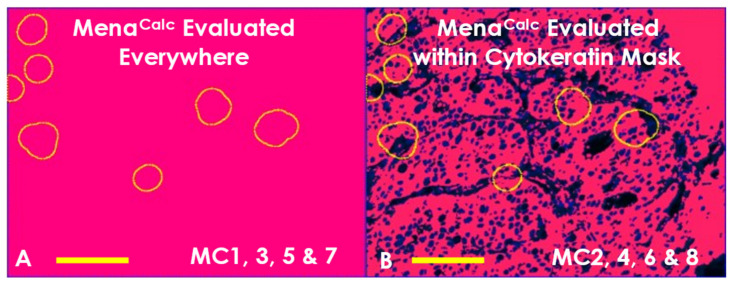
Additional Sub Types of Mena^Calc^ Analyses. Mena^Calc^ was evaluated within the same four areas as was TMEM doorway. In addition, Mena^Calc^ quantification was further separated into two subcategories by either applying a cytokeratin mask to limit the quantification to extra-nuclear tumor cells or not. (**A**) Mena^Calc^ Evaluated Everywhere. This type of analysis measures Mena signals within the entire image including both nuclear and extra-nuclear. TMEM doorways were circled in yellow. Scale bar = 100 µm. (**B**) Mena^Calc^ Evaluated within Cytokeratin Mask. This type of analysis only measures signal within a mask determined by cytokeratin staining. TMEM doorways were circled in yellow. Scale bar = 100 µm.

We evaluated Mena^Calc^ using the same approaches described above. In addition, in the original Mena^Calc^ publications [21,22], quantification of Mena signals was limited to a binary “tumor mask” representing only epithelial cells and excluding stromal features. To determine if this signal was indeed important for the performance of the marker, we additionally varied whether the Mena^Calc^ was measured within the entire image (Figure 3A, pink area), or just within the area determined by a thresholded cytokeratin signal mask (Figure 3B, pink area). As a result, eight different variations of Mena^Calc^ were evaluated, named MC1 through MC8 (Table 1).

While this creates many variations for the Combined Marker (8 × 4 = 32 combinations), we only considered combinations of TMEM and Mena^Calc^ scores that utilized the same ROI type and tissue coverage for both markers. This left only the eight combinations indicated in Table 2.

### 2.2. Measurement of Performance

**Figure 4:**
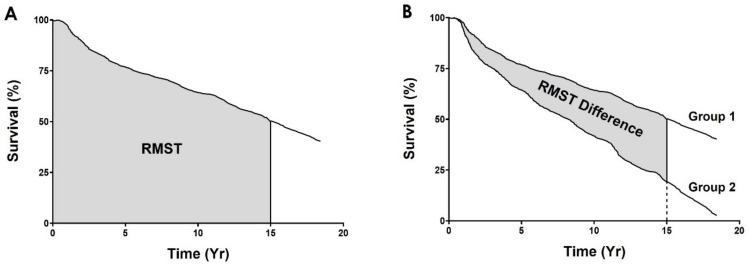
Graphic representation of restricted mean survival time and its difference. (**A**) RMST is the integration of survival probability across a pre-specified time, which graphically is the area under the survival curve. (**B**) RMST difference represents the group separation of the survival analysis.

The performance of each individual marker was determined by establishing a cut-off point which divided the cohort into “Low” and “High” score groups. The progression-free survival curves of these groups were then compared using Kaplan–Meier analyses, which uses disease progression as an endpoint, i.e., the development of distant metastasis in this study [23]. In order to quantify the separation between survival curves, we employed the restricted mean survival time (RMST) difference. RMST is a well-established method for evaluating survival data in clinical trials [24,25,26,27,28,29,30,31,32]. This metric quantifies the average event-free survival time, up to (or restricted to) a pre-specified, clinically important time point, which corresponds graphically to the area under the survival curve [33] (Figure 4A). The absolute difference in RMST between two study groups thus provides an estimate of the event-free life expectancy difference between the groups [32]. Graphically, it is the difference between the areas under the survival curves (Figure 4B), i.e., the group separation.

**Figure 5:**
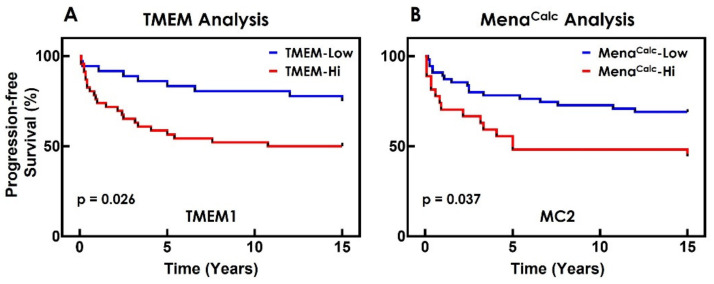
Progression-free survival curves for TMEM doorway and Mena^Calc^ analyses. Distant metastasis of breast tumor was used as the endpoint in this study. (**A**) Kaplan–Meier curve for the TMEM doorway analysis in Whole-Tumor Tissue ROI, Entire Area (TMEM cut-off point = 5.16). (**B**) Kaplan–Meier curve for the Mena^Calc^ analysis in Whole-Tumor Tissue ROI, Entire Area, with Cytokeratin Mask (Mena^Calc^ cut-off point = 0.02).

Thus, in order to determine the best possible prognostic performance of each marker individually, we varied the cut-off point over the range of possible values to establish the optimal cut-off point, i.e., the cut-off point value which produced the best group separation (Figure 5A,B; Figure S2).

Since the RMST difference calculation depends upon having two survival curves, the calculation breaks down when all patients fall into a single group. Furthermore, the RMST difference calculation may produce artificially high differences when a group contains only a few patients whose survival time is very short. Observing very few patients in either group would be an unrealistic situation since it is expected that ~20–40% of ER+ patients would experience distant metastasis [34]. Thus, we limited the range of the cut-off points so that the “Low” and “High” groups both contained at least 10% of the entire cohort size.

To construct a Combined Marker, we used the logical “AND” operation between the two best-performing TMEM doorway and Mena^Calc^ tests so that cases were considered “High” for the Combined Marker if they were “High” for both the TMEM doorway and Mena^Calc^ markers individually, and “Low” for the Combined Marker if they were “Low” for at least one (Figure S4). Given the small cohort size of this study, we limited the analysis to just two groups. To objectively evaluate the comparison between the performance of the Combined Marker and that of the individual tests, cut-off points were not altered from their optimal values. This prevents “over-training” of the system and allows evaluation of the increase in performance of the Combined Marker over the best possible performance of each individual marker alone. The RMST difference values and cut-off points for all analyses are tabulated in Table 3.

Among the eight Combined Marker analyses, five divided the two groups with less than 10% of the population in one group (Table 4). All three of the remaining analyses showed improved differentiation in progression-free survival compared to their respective TMEM doorway and Mena^Calc^ analyses alone (Table 3).

### 2.3. Determination of Best Performer

**Figure 6:**
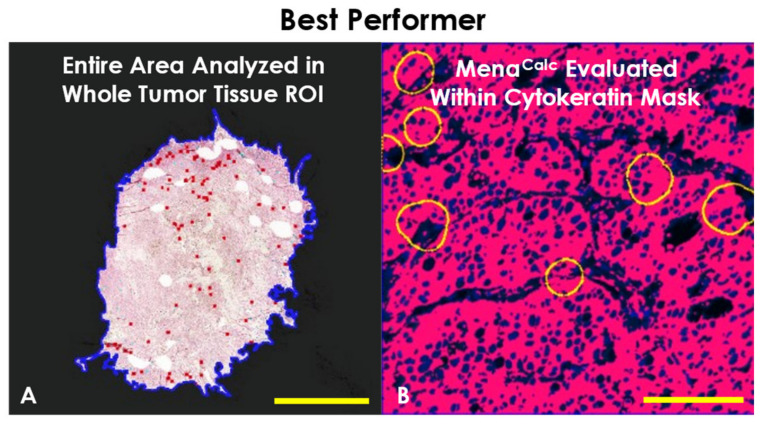
Performing Combined Marker analysis. The Combined Marker analysis which showed the highest performance gain over both TMEM doorway and Mena^Calc^ alone was the one evaluated in the entire area of the Whole-Tumor Tissue ROI (**A**) and utilized cytokeratin mask in the Mena^Calc^ evaluation (**B**). TMEM doorways were circled in yellow. Scale bar in (**A**) = 5 mm. Scale bar in (**B**) = 100 µm.

**Figure 7:**
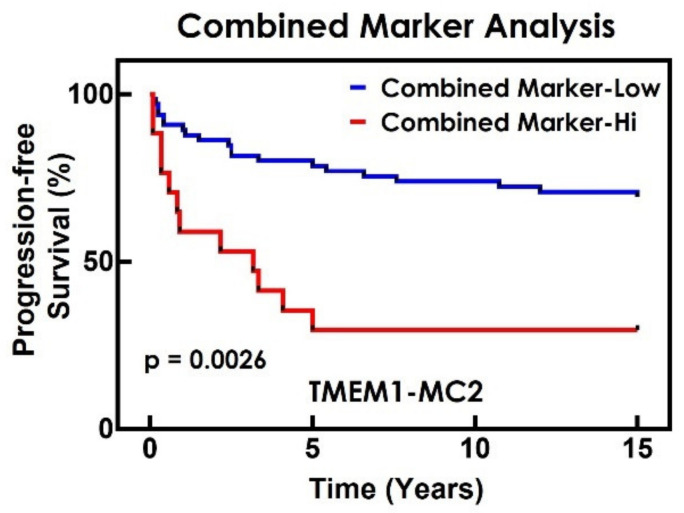
Progression-free survival curves for best-performing Combined Marker. Distant metastasis of breast tumor was used as the endpoint in this study. Kaplan–Meier curve for the best performing Combined Marker analysis (TMEM cut-off point = 5.16, Mena^Calc^ cut-off point = 0.02).

The analysis which resulted in the largest RMST difference value was TMEM1-MC2, the test where the TMEM Score and the Mena^Calc^ Score were evaluated over the entire area of the whole tumor tissue ROI (Figure 6A) and utilized a cytokeratin mask to limit the Mena^Calc^ evaluation to tumor cell cytoplasm (Figure 6B). TMEM1-MC2 gave an RMST difference of 5.27 years (95% CI: 1.71–8.37), compared to 3.56 years (95% CI: 0.95–6.1) for TMEM1 and 2.94 years (95% CI: 0.25–5.87) for MC2, i.e., 1.71 years longer in progression-free survival than TMEM1 alone and 2.37 years longer than MC2 alone. Despite the large and overlapping confidence intervals, this Combined Marker analysis shows a markedly improved ability to discriminate between those patients who experience distant recurrence and those who do not (Figure 7). The number of patients in each individual risk group is shown in Table 5.

## 3. Discussion

Our study of the different methods of combining TMEM doorway and Mena^Calc^ analyses showed a noticeable improvement in prognostic performance when measuring TMEM doorways and Mena^Calc^ over the entire range of the tumor tissue and utilizing a cytokeratin mask to limit the Mena^Calc^ evaluation to tumor cell cytoplasm only. This result can be understood by considering the interaction between tumor cell intravasation sites (TMEM doorways) and highly motile (Mena^Calc-Hi^) tumor cells (Figure 1D).

Using high-resolution intravital imaging, we have previously shown that, during intravasation, tumor cells respond to chemotactic signals, migrate towards blood vessels, and enter the blood stream through TMEM doorways [6,7,14]. Tumor cells within a 500 µm radius of blood vessels are attracted towards the blood vessels by hepatocyte growth factor (HGF) gradients [14]. In tumor cells lying between 500 µm and 1000 µm from blood vessels, migration is not directed towards the blood vessels but is toward macrophages which draws the tumor cell macrophage pairs into the HGF gradient. The tumor-cell–macrophage paired chemotaxis is driven by a paracrine loop between tumor-cell-secreted colony-stimulating factor 1 (CSF-1) and macrophage-secreted epidermal growth factor (EGF) [13]. Both chemotaxis-mediated tumor cell movements are greatly amplified in tumor cells with high Mena^Calc^ levels [14,16,17,18]. Thus, there may be a high probability of tumor cell intravasation when Mena^Calc-Hi^ cells are close (within an area ~1 mm in radius) to blood vessels that contain TMEM doorways (Figure 1A). By centering this area upon each TMEM doorway, we can define a “TMEM interaction zone” wherein Mena^Calc-Hi^ tumor cells are likely to intravasate.

In patients with a high TMEM Score, the density of TMEM doorways is high enough that the interaction zone for one TMEM doorway may overlap with that of adjacent TMEM doorways and thus the total interaction zone may cover the tumor volume nearly completely. Therefore, the improved performance when the Combined Marker is evaluated over the entire range of the tumor tissue (vs. just 10 fields of view) is to be expected as a result of increased sampling of the tissue which has the net effect of “averaging out” tissue heterogeneity.

Furthermore, the improved performance of the Combined Marker when utilizing the cytokeratin mask is to be expected as well, for two reasons. The first is that a cytokeratin mask limits the signal to only the tumor cells and excludes non-specific binding of antibody to stromal cells. Secondly, since Mena is a cytoplasmic and membrane-bound protein, exclusion of nuclei narrows the area for analysis to only the one where Mena is expected to be found. Both of these effects improve the specificity of detection and better separate signal from noise.

## 4. Materials and Methods

### 4.1. Cohort

Patient cases were taken from the Calgary Tamoxifen cohort (described previously in [35]), a large retrospective cohort of breast cancer patients diagnosed between 1985 and 2000. A subset of 130 patients (randomly chosen based on recurrence status, 65 patients with no distant recurrence and 65 patients with a distant recurrence at 5 years) that had previously been utilized to investigate the influence of ATM protein in both tumor and cancer-associated stroma on clinical outcome [36] was used for this study. The information regarding overall survival was not available at the time of the study. Patients had confirmed invasive breast cancer (74 ductal, 8 lobular, 2 other, 1 unknown) and received adjuvant tamoxifen therapy. Patients were excluded from this analysis if they had a prior cancer diagnosis (except non-melanoma skin cancer). Because most patients with ER+ disease do not show additional benefits from chemotherapy compared to endocrine therapy alone [37,38], and it is expected that most patients with ER+ breast cancer will be treated with endocrine therapy alone, patients who received neo- or adjuvant-chemotherapy were also excluded from the study. Moreover, chemotherapy may increase TMEM Score in some patients with ER+ disease [39]. In addition, it should be noted that chemoendocrine therapy in node-positive ER+ breast cancer patients was shown to be beneficial in pre-menopausal women (RxPONDER trial), and is continued to be included as standard of care treatment option for these patients. Of the 130 patients, pathological review determined that 86 cases had sufficient tissue for analysis. The characteristics of the study cohort are summarized in Table 6. The maximum follow-up time is 15 years.

### 4.2. IHC Triple Staining

TMEM IHC staining was carried out by a commercial entity (MetaStat, Boston, MA, USA) using the MetaSite Breast™ assay which measures TMEM Score as described previously [10]. According to the company, formalin-fixed paraffin-embedded invasive breast cancer samples were stained for TMEM doorways using a modified triple chromogen immunohistochemical stain for CD31-positive blood vessels using a rabbit anti-CD31 monoclonal antibody (AbCam/Epitomics Clone EP3095, Burlingame, CA, USA), CD68-positive macrophages using an anti-CD68 mouse monoclonal antibody (Thermo Scientific Clone PGM1, Waltham, MA, USA), and Mena-positive tumor cells using a proprietary anti-PanMena mouse monoclonal antibody. CD31-positive blood vessels, CD68-positive macrophages, and Mena-expressing tumor cells were visualized using brown, blue, and red chromogens, respectively.

**Table 6:** Clinical–pathological characteristics of patient cohort. For the final analysis, 86 out of the 130 total patient cases were included.

		Full Cohort		Analyzed vs. Not		15 YR Disease-Free Survival	
		# of Cases	%	Not Analyzed	Analyzed	No Event	Event
		(n = 130)		(n = 44)	(n = 86)	(n = 43)	(n = 43)
**AGE**							
	Range	37.9–88.1					
	Median	70.1					
	<53	18	13.9	6	12	10	2
	≥53	112	86.2	38	74	33	41
**TUMOUR SIZE**							
	Range	0.4 cm–13.0 cm					
	Median	2 cm					
	<2 cm	53	40.8	14	39	26	13
	≥2 cm	73	56.2	28	45	17	28
	Unknown	4	3.1	2	2	0	2
**GRADE**							
	Low (1/2)	88	67.7	31	57	32	25
	High (3)	32	24.6	8	24	10	14
	Unknown	10	7.7	5	5	1	4
**NODE STATUS**							
	Negative	61	46.9	20	41	27	14
	Positive	49	37.7	16	33	10	23
	Unknown	20	15.4	8	12	6	6
**ER STATUS**							
	Negative	5	3.9	0	5	0	5
	Positive	100	76.9	22	78	40	38
	Unknown	25	19.2	22	3	3	0
**PR STATUS**							
	Negative	12	9.2	1	11	3	8
	Positive	87	66.9	18	69	34	35
	Unknown	31	23.9	25	6	6	0
**HER2 STATUS**							
	Negative	106	81.5	23	83	41	42
	Positive	4	3.1	1	3	2	1
	Unknown	20	15.4	20	0	0	0

### 4.3. Multiplexed Immunofluorescence Staining

Mena^Calc^ immunofluorescence staining was also done by the commercial entity (MetaStat, Boston, MA, USA). According to the company, staining was performed using a modified indirect multiplexed fluorescence assay [21,22] via HRP (ImmPRESS Reagents, Vector Laboratories, Burlingame, CA, USA)/Tyramide (Life Technologies, Carlsbad, CA, USA) signal amplification to stain for PanMena using an anti-PanMena mouse monoclonal antibody (Gertler Laboratory, MIT, Cambridge, MA, USA; 0.05 µg/mL) and Mena11a using an anti-Mena11a rabbit polyclonal antibody (Gertler Laboratory, MIT; 3.3 µg/mL). Epithelial cells were identified using a pan-Cytokeratin mouse monoclonal antibody directly conjugated to an Alexa488 fluorophore (EBiosciences, Thermo Fisher Scientific, Waltham, MA, USA). Mena and Mena11a were visualized using Alexa555 TSA and Alexa647 TSA, respectively. Nuclei were visualized with DAPI.

### 4.4. Digital Whole Slide Scanning

Slides were digitized on the PerkinElmer Pannoramic 250 Flash II digital whole-slide scanner using a 20x 0.8NA Plan-Apochromat objective (PerkinElmer, Hopkinton, MA, USA). A typical digital whole-slide scan consists of thousands of fields which are mosaicked to form a high-resolution image for analysis. TMEM doorway slides were imaged in bright field mode (pixel size = 0.24 µm, bit depth = 8) and Mena^Calc^ slides were imaged in fluorescence mode (pixel size = 0.33 µm, bit depth = 8).

### 4.5. Automated TMEM Doorway Quantification

After scanning, the digital slides were imported into Visiopharm’s image analysis software, Vis (Visiopharm, Hørsholm, Denmark). In Vis, Mena^Calc^ slides were aligned to TMEM doorway slides using the Tissue Align module. The boundaries of each tissue were determined by heavily smoothing (51 × 51 px kernel) the negated brightfield image of the TMEM doorway stained slide and thresholding the resulting signal (“Whole Tumor Tissue ROI”, Figure 2A). Next, three pathologists identified more limited regions of interest (“Path ROIs”, Figure 2B) for analysis based upon appropriate pathological criteria (e.g., high density of blood vessels, low levels of stromal tissue) and image quality (e.g., out of focus regions). In addition, staining quality and adequacy of tissue on both TMEM doorway and IF-stained slides were checked in all ROIs.

The 10 highest TMEM doorway scoring high power fields were identified using an automatic ranking mechanism as previously published [5,10]. Briefly, the MicroImager module in Vis divided the entire area of Whole Tumor Tissue or the Path ROIs into subfields of equal area with each subfield equivalent to a pathologist’s microscope high-power field (330 × 440 µm^2^ each, Figure 2G). Next, TMEM doorways were quantified in all of the high-power fields and the 10 fields with the highest number of TMEM doorways were identified and the sum of TMEM doorways in these fields was used as the TMEM Score for the patient sample (Top 10 TMEM Score) [5].

### 4.6. Automated Mena^Calc^ Quantification

Mena^Calc^ was quantified similarly to previous publications as the difference between PanMena and Mena11a z-scores (Equations (1)–(3)) (Figure S3) [21,22]. Following TMEM doorway quantification, Mena^Calc^ was measured within the ROIs and tissue coverage area as described in Table 1 and Figure 2. In addition, Mena^Calc^ quantification was further measured across all cells (Figure 3A) or restricted to only the area with a mask generated by a cytokeratin stain (Figure 3B). This resulted in a total of eight different methods of Mena^Calc^ quantification (Table 1).

(1) $${\mathrm{Mena}}^{\mathrm{Calc}}\;=\;\mathrm{PanMena}\;Z\;-\mathrm{Mena}11a\;Z$$

(2) $$\mathrm{PanMena}\;Z\;=\left(x\;-{\;\mu }_{\mathrm{PanMena}}\right)/\sigma_{\mathrm{PanMena}}$$

(3) $$\mathrm{Mena}11a\;Z\;=\left(x\;-{\;\mu }_{\mathrm{Mena}11a}\right)/\sigma_{\mathrm{Mena}11a}$$

### 4.7. Statistical and Survival Analysis

All statistical analyses were performed and automated using R 4.0.4 in RStudio (RStudio, Inc., Boston, MA, USA).

### 4.8. Standalone TMEM Doorway and Mena^Calc^ Analyses

In each of the four TMEM doorway tests (Table 1), patient cases were separated into two risk groups (high and low risk) by a cut-off point value so that the TMEM Score of the high-risk group was equal to or greater than the cut-off point value and the TMEM Score of the low risk group was less than the cut-off point value. A custom-written R script was used to automate the analysis by incrementally varying the cut-off point value across the range of the TMEM scores (60 equally spaced cut-off points were used), constructing a Kaplan–Meier survival curve, and evaluating the group separation with a RMST difference value for each cut-off point. Only the analyses which generated both high-risk and low-risk group sizes greater than 10% of the population were recorded. The optimal cut-off point value for the TMEM doorway analysis was chosen as the cut-off point which generated the maximum RMST difference value within the series. Bootstrapping with 1000 repetitions was performed to estimate confidence intervals for the optimal cut-off point.

The eight standalone Mena^Calc^ analyses were performed in a similar way to the four TMEM doorway analyses.

### 4.9. Combined Marker Analysis

For the Combined Marker analyses, a TMEM doorway test and a Mena^Calc^ test were paired to form a combined test pair (Table 2). Among the total 32 possible Combined Marker analyses, only 8 were consistent in the range and quantity of tissue analyzed (Table 2). In the Combined Marker analysis, a patient was included in the high-risk group only if they fell within the high-risk group in both the associated TMEM doorway and Mena^Calc^ analyses (Figure S4). Otherwise, they were deemed low risk. In each Combined Marker analysis, the TMEM cut-off point and Mena^Calc^ cut-off point were taken directly from the corresponding standalone TMEM doorway analysis and Mena^Calc^ analysis. Only the analyses which generated both group sizes greater than 10% of the population were recorded. The RMST difference values and cut-off point values of the Combined Marker analyses and the associated TMEM doorway and Mena^Calc^ analyses are given in Table 3. The entire Combined Marker analysis was also automated in a custom-written R script.

## 5. Conclusions

In conclusion, we have developed and evaluated a potential new biomarker for prognosticating metastatic progression in ER+/HER2− breast cancer patients that combines the previously published TMEM doorway and Mena^Calc^ prognostic biomarkers. Our results show that the Combined Marker potentially improves prognostication over that observed with TMEM or Mena^Calc^ Score alone. While promising, the patient cohort in this study is limited in size, only considers HR+/HER2− breast cancer, and lacks an independent validation cohort to confirm the results. Future work currently in progress is focused on validating these results in a larger, independent cohort (including HER2 patients), expanding the analysis to include other breast cancer subtypes and, importantly, accounting for other variables with a multivariate analysis.

## Figures and Tables

**Table 1:** Types of TMEM doorway and Mena^Calc^ analyses. Description of the four different methods of TMEM doorway analyses and eight different Mena^Calc^ Analyses. MC stands for Mena^Calc^. TMEM doorway analyses vary in ROI type and tissue coverage, which gives four TMEM doorway quantifications. Aside from the two variations in TMEM doorway analyses, Mena^Calc^ analyses also vary in tissue type, i.e., tumor or stroma, differentiated by a cytokeratin mask, which in total gives eight Mena^Calc^ analyses.

Quantification Parameters			
Method	ROI Type	Tissue Coverage	Cytokeratin Mask
**TMEM1**	Whole Tumor Tissue	Entire Area	
**TMEM2**	Path ROI	Entire Area	
**TMEM3**	Whole Tumor Tissue	Top 10 Fields	
**TMEM4**	Path ROI	Top 10 Fields	
**MC1**	Whole Tumor Tissue	Entire Area	No
**MC2**	Whole Tumor Tissue	Entire Area	Yes
**MC3**	Path ROI	Entire Area	No
**MC4**	Path ROI	Entire Area	Yes
**MC5**	Whole Tumor Tissue	Top 10 Fields	No
**MC6**	Whole Tumor Tissue	Top 10 Fields	Yes
**MC7**	Path ROI	Top 10 Fields	No
**MC8**	Path ROI	Top 10 Fields	Yes

**Table 2:** Valid combined marker test pairs. While the variation of ROI type, tissue coverage, and application of a cytokeratin mask creates 32 potential combinations of TMEM and Mena^Calc^ scores, only eight were evaluated within the same ROI type and tissue coverage. Thus, Combined Marker was evaluated in these 8 valid combinations.

Combined Marker Test Pairs				
Method	TMEM1	TMEM2	TMEM3	TMEM4
**MC1**	TMEM1-MC1			
**MC2**	TMEM1-MC2			
**MC3**		TMEM2-MC3		
**MC4**		TMEM2-MC4		
**MC5**			TMEM3-MC5	
**MC6**			TMEM3-MC6	
**MC7**				TMEM4-MC7
**MC8**				TMEM4-MC8

**Table 3:** RMST Difference Values for TMEM Doorway, Mena^Calc^, and Combined Marker Analyses. Upper table gives RMST difference values (and their associated confidence intervals) for each of the four TMEM doorway analyses and the associated TMEM cut-off point values, which stratify the patients into high- and low-risk groups (i.e., high TMEM Score means high risk, and low TMEM Score means low risk). Likewise, left table gives RMST difference values (and their associated confidence intervals) for each of the eight Mena^Calc^ analyses and the associated Mena^Calc^ cut-off point values, which stratify the Mena^Calc^ scores into high- and low-risk groups. The main table gives RMST difference values (and their associated confidence intervals) for the available valid Combined Marker analyses in which a patient must have both high TMEM Score and high Mena^Calc^ Score to be considered as high risk. Only the analyses which generated both group sizes containing greater than 10% of the population were recorded.

			**TMEM RMST Difference**				
			**Method**	**TMEM1**	**TMEM2**	**TMEM3**	**TMEM4**
			**Cut Point**	5.16	14.51	38.08	77.95
			**RMST Difference (Yr)**	3.56 (95% CI: 0.95–6.1)	4.57 (95% CI: 1.73–7.08)	4 (95% CI: 1.42–6.59)	3.56 (95% CI: −0.4–7.26)
**Mena^Calc^ RMST Difference**			**Combined Marker RMST Difference**				
**Method**	**Cut Point**	**RMST Difference (Yr)**	**Method**	**TMEM1**	**TMEM2**	**TMEM3**	**TMEM4**
**MC1**	0.44	0.89 (95% CI: −2.61–4.75)	**MC1**	NA			
**MC2**	0.02	2.94 (95% CI: 0.25–5.87)	**MC2**	5.27 (95% CI: 1.71–8.37)			
**MC3**	−0.01	2.46 (95% CI: −0.19–5.22)	**MC3**		5.16 (95% CI: 1.68–8.77)		
**MC4**	0.35	0.97 (95% CI: −2.91–5.14)	**MC4**		NA		
**MC5**	−0.31	1.06 (95% CI: −1.84–3.88)	**MC5**			4.53 (95% CI: 1.73–7.08)	
**MC6**	0.38	5.32 (95% CI: 1.04–8.94)	**MC6**			NA	
**MC7**	0.21	1.66 (95% CI: −1.38–4.65)	**MC7**				NA
**MC8**	0.34	2.29 (95% CI: −1.83–6.62)	**MC8**				NA

**Table 4:** Number of patients in the high-risk group for TMEM Doorway, Mena^Calc^, and Combined Marker analyses. A cut-off point value stratifies the patients into high and low risk groups. In Combined Marker analyses, the grouping tends to be skewed towards the low risk group.

		**# of Patients in TMEM-Hi**				
		**Method**	**TMEM1**	**TMEM2**	**TMEM3**	**TMEM4**
		**# of Patients**	48	37	49	12
**# of Patients in Mena^Calc^-Hi**		**# of Patients in Combined Marker-Hi**				
**Method**	**# of Patients**	**Method**	**TMEM1**	**TMEM2**	**TMEM3**	**TMEM4**
**MC1**	12	**MC1**	4			
**MC2**	28	**MC2**	17			
**MC3**	36	**MC3**		15		
**MC4**	12	**MC4**		5		
**MC5**	62	**MC5**			32	
**MC6**	11	**MC6**			7	
**MC7**	26	**MC7**				2
**MC8**	10	**MC8**				1

**Table 5:** Number of patients in each individual risk group for best-performing Combined Marker. A total of 17 out of 86 cases were high in both TMEM and Mena^Calc^ scores, which were then considered high risk in Combined Marker analysis (TMEM cut-off point = 5.16, Mena^Calc^ cut-off point = 0.02).

# of Cases	TMEM-Hi	TMEM-Low
**Mena^Calc^-Hi**	17	11
**Mena^Calc^-Low**	31	27

## Data Availability

The data presented in this study are available on request from the corresponding author.

## Supplementary Materials

The following supporting information can be downloaded at: https://www.mdpi.com/article/10.3390/cancers14092168/s1, Figure S1: Representative Histology Slides of TMEM, PanMena, and Mena11a from Combined Marker-Hi and Combined Marker-Low Patients; Figure S2: The Establishment of Optimal Cut-off Point for TMEM doorway/Mena^Calc^ Analysis; Figure S3: Visual Representation of Mena^Calc^ in Terms of PanMena and Mena11a Intensities; Figure S4: Visual Representation of Combined Marker in Terms of TMEM Score and Mena^Calc^ Score.
