# Supplementary material for: Combining TMEM Doorway Score and MenaCalc Score Improves the Prediction of Distant Recurrence Risk in HR+/HER2− Breast Cancer Patients
Source: Cancers (Basel). 2022 Apr 26;14(9):2168. doi: 10.3390/cancers14092168 (PMC9101795; doi:10.3390/cancers14092168)
Supplement: Supplementary file 1 [file cancers-14-02168-s001.zip › cancers-1607677 supplementary.pdf]

Supplemental Materials

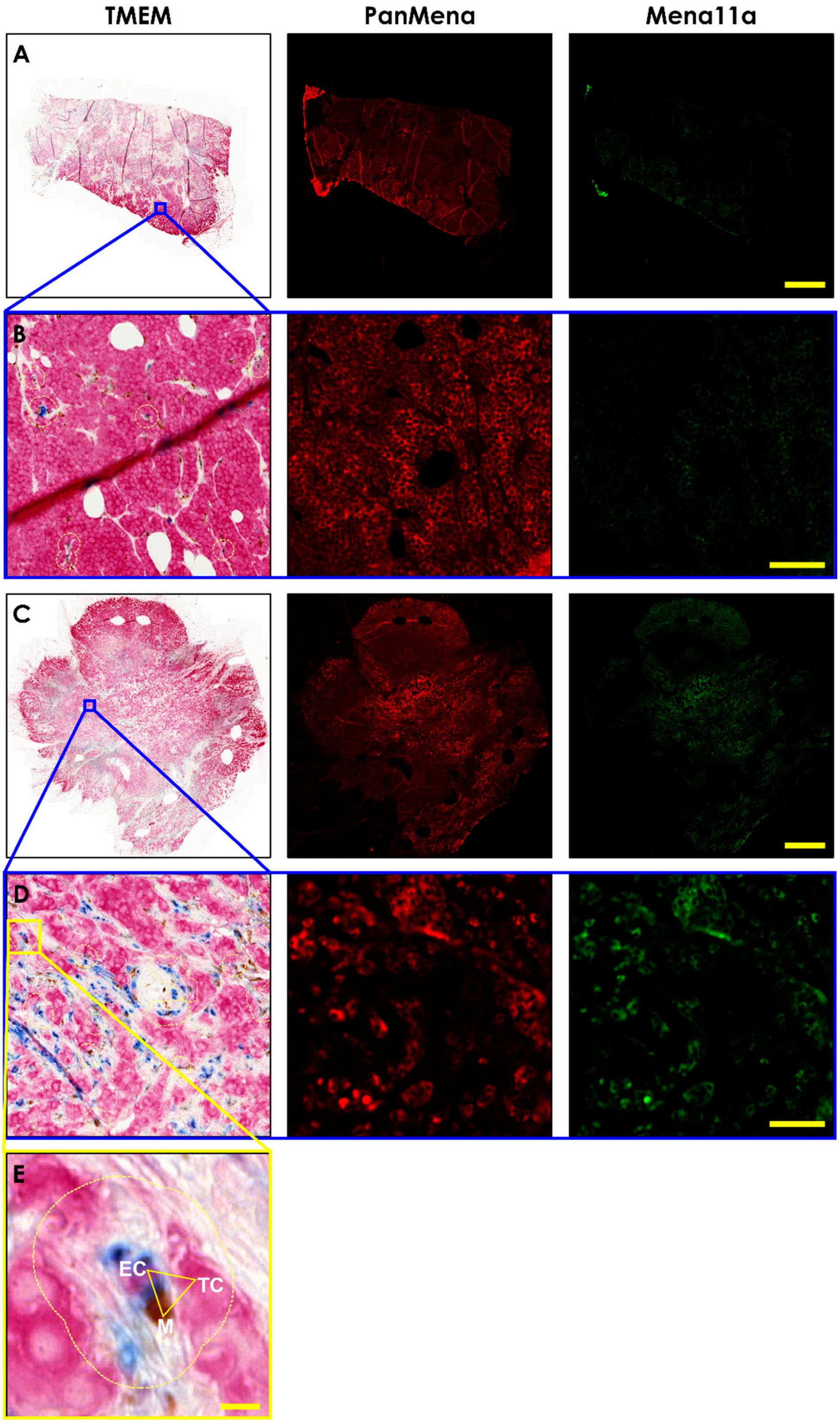

Figure S1. Representative Histology Slides of TMEM, PanMena, and Mena11a from Combined Marker-Hi and Combined Marker-Low Patients. **A)** Example images of stained IHC (TMEM) and IF (PanMena and Mena11a) slides from a Combined Marker-Hi patient with both high TMEM Score and $Mena^{Calc}$ Score. Left: TMEM (Pink - Mena overexpressing tumor cell; Blue – Endothelial cell; Brown – Perivascular macrophage). Middle: PanMena in red channel. Right: Mena11a in green channel (pseudo color). The difference between PanMena and Mena11a intensities indicates a higher amount of pro-metastatic Mena isoforms, resulting in a $Mena^{Calc}$-Hi score. Scale bar = 2,000 μm. **B)** Zoomed in view of **A**. Left: TMEM doorways are circled in yellow. Scale bar = 100 μm. **C)** Example images of stained IHC (TMEM) and IF (PanMena and Mena11a) slides from a Combined Marker-Low patient with high TMEM Score and Low $Mena^{Calc}$ Score. Similar PanMena and Mena11a intensities indicates a lower amount of pro-metastatic Mena isoforms, which results in a $Mena^{Calc}$-Low score. Scale bar = 2,000 μm. **D)** Zoomed in view of **C**. Left: TMEM doorways are circled in yellow. Scale bar = 100 μm. **E)** A representative IHC image of TMEM doorway. TC: Tumor Cell. EC: Endothelial Cell. M: Macrophage. Scale bar = 10 μm.

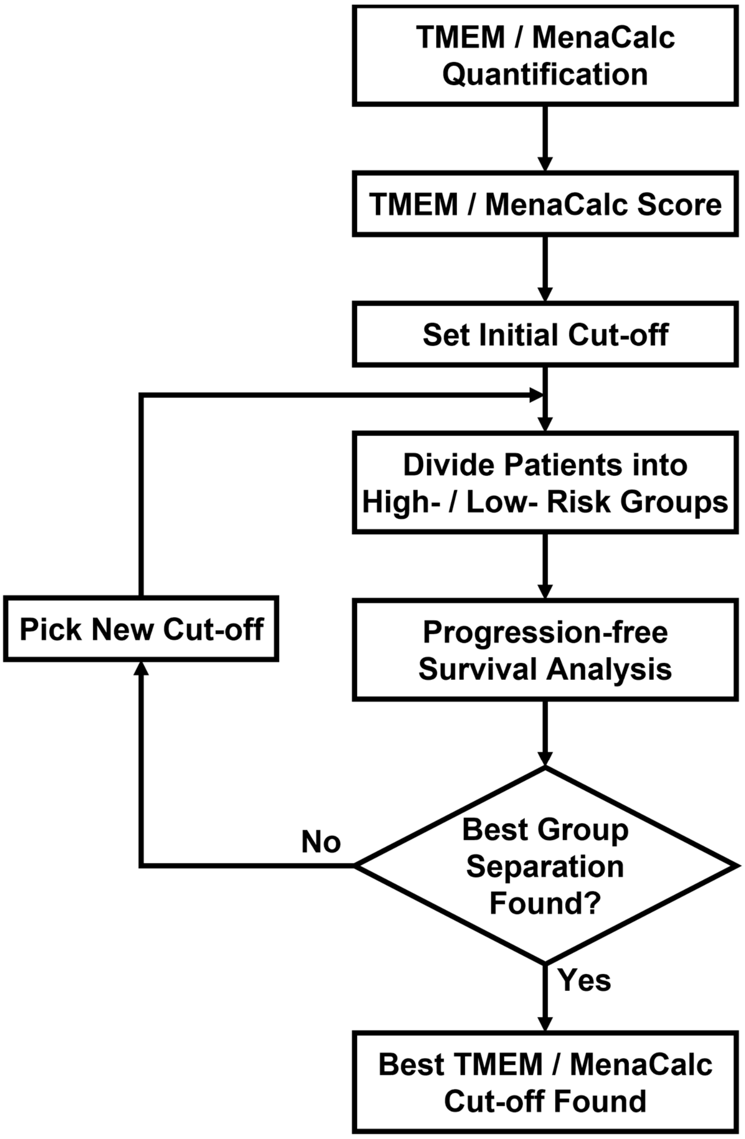

Figure S2. The Establishment of Optimal Cut-off Point for TMEM doorway/Mena^{Calc} Analysis. The cut-off point was varied over the range of possible values to establish the optimal cut-off point value which produced the best group separation.

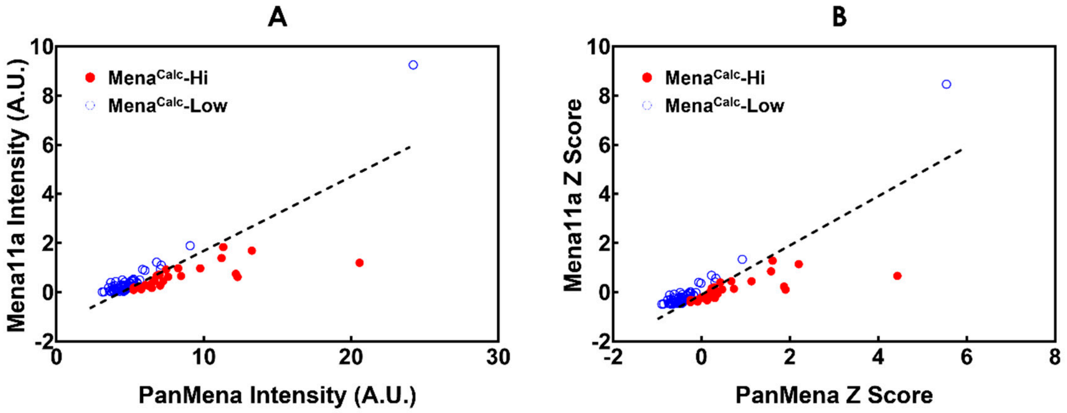

C

$$\mathbf{Mena^{Calc}} = \text{PanMena Z} - \text{Mena11a Z}$$

$$\text{PanMena Z} = \frac{x - \mu_{\text{PanMena}}}{\sigma_{\text{PanMena}}}$$

$$\text{Mena11a Z} = \frac{x - \mu_{\text{Mena11a}}}{\sigma_{\text{Mena11a}}}$$

Figure S3. Visual Representation of Mena^{Calc} in Terms of PanMena and Mena11a Intensities. **A)** Mena11a intensity and PanMena intensity. The dashed line separates the patients into two groups. Above the line, patient samples show lower PanMena intensity and higher Mena11a intensity, which implies lower amount of pro-metastatic Mena isoforms, thus Mena^{Calc}-Low. Below the line, patient samples show higher PanMena intensity and lower Mena11a intensity, which implies higher amount of pro-metastatic Mena isoforms, thus Mena^{Calc}-Hi. **B)** Normalized Mena11a intensity and normalized PanMena intensity are used directly to compute Mena^{Calc} Score. **C)** Definition of Mena^{Calc} in terms of PanMena and Mena11a intensities. x: fluorescence intensity of PanMena or Mena11a. μ: sample mean. σ: standard deviation.

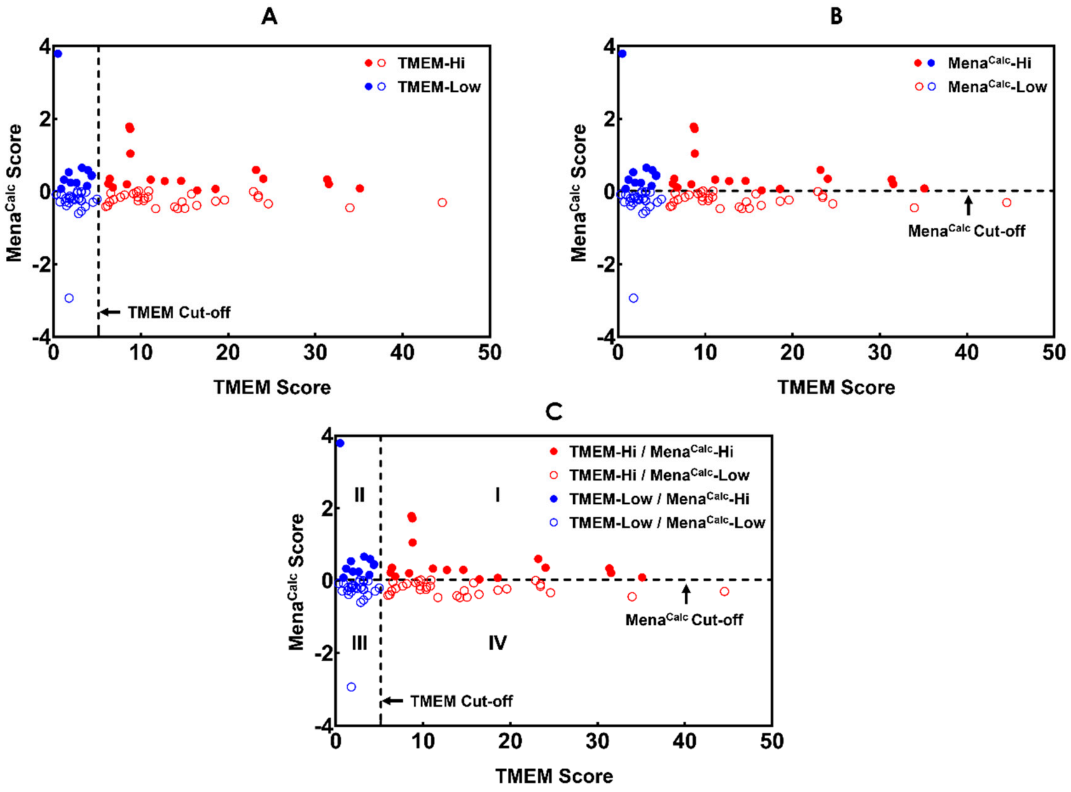

Figure S4. Visual Representation of Combined Marker in Terms of TMEM Score and Mena^{Calc} Score. **A)** A TMEM cut-off point (5.16), represented by the vertical dashed line, separates patient cohort into TMEM-Hi (dots and circles in red) and TMEM-Low (dots and circles in blue) risk groups, regardless of Mena^{Calc} Score. **B)** Likewise, a Mena^{Calc} cut-off point (0.02) (horizontal dashed line) separates patients into Mena^{Calc}-Hi (dots in red and blue) and Mena^{Calc}-Low (circles in red and blue) risk groups, regardless of TMEM Score. **C)** By combining TMEM doorway and Mena^{Calc} analyses, patients are sorted into four groups, defined by the TMEM and Mena^{Calc} cut-off lines. Only patients with both high TMEM Score and high Mena^{Calc} Score (Quadrant I) are considered Combined Marker-Hi. All the other patients are Combined Marker-Low (Quadrants II to IV).
